# DMA^V^ in Drinking Water Activated NF-*κ*B Signal Pathway and Increased TGF-*β* and IL-1*β* Expressions in Bladder Epithelial Cells of Rats

**DOI:** 10.1155/2015/790652

**Published:** 2015-11-05

**Authors:** Siqi Cao, Shengnan Liu, Fei Wang, Jieyu Liu, Mengdan Li, Chen Wang, Shuhua Xi

**Affiliations:** Department of Environmental and Occupational Health, School of Public Health, China Medical University, No. 77 Puhe Road, Shenyang North New Area, Shenyang, Liaoning 110122, China

## Abstract

Dimethylarsinic acid (DMA^V^) is the main product of arsenic methylation metabolism in vivo and is rat bladder carcinogen and tumor promoting agent. In this study, we measured the expressions of mRNA and proteins of NF-*κ*B pathway members, IKK*α*, IKK*β*, p65, and p50 in rat bladder epithelium by qRT-PCR and immunohistochemical analysis after rats received drinking water containing 100 and 200 ppm DMA^V^ for 10 weeks. Transforming growth factor-*β* (TGF-*β*) immunoexpression in rat bladder epithelium and urine level of IL-1*β* also were determined. We found that DMA^V^ dramatically increased the mRNA levels of NF-*κ*B p50 and IKK*α* in the bladder epithelium of rats compared to the control group. Immunohistochemical examinations showed that DMA^V^ increased immunoreactivities of IKK*α*, IKK*β*, and phospho-NF-*κ*B p50 in the cytoplasm and phospho-NF-*κ*B p50 and p65 in nucleus of rat urothelial cells. In addition, DMA^V^ treated rats exhibited significantly increased inflammatory factor TGF-*β* immunoreactivity in bladder epithelium and IL-1*β* secretion in urine. These data suggest that DMA^V^ could activate NF-*κ*B signal pathway and increase TGF-*β* and IL-1*β* expressions in bladder epithelial cells of rats.

## 1. Introduction

Dimethylarsinic acid (DMA^V^) is the main product of arsenic methylation metabolism in vivo, excreted in urine. The animal experiment showed that rat exposure to DMA^V^ suffered from bladder toxicity, especially urothelial tumors [1–3].  DMA^V^ is considered to be the rat bladder carcinogen and tumor promoting agent [4]. The mechanism of DMA^V^ induced cancer may be related to proliferation, apoptosis, oxidative stress, and inflammatory reaction. Our previous study showed that DMA^V^ increased the expressions of proliferation factors in bladder urothelium and elevated transforming growth factor-beta 1 (TGF-*β*1) secretion and decreased tumor necrosis factor-alpha (TNF-*α*) level in the urine of rats [5]. Our and other studies suggested that chronic inflammation, bladder epithelium lesions, and proliferation might be the basic process of the chronic toxicity effects of DMA^V^ on rats.

It was reported that nuclear factor-kappa B (NF-*κ*B) nuclear expression is correlated with histologic grade and T category in bladder urothelium cancer [6]. NF-*κ*B is a heterodimeric, sequence-specific transcription factor and consists of two major subunit polypeptides, p50 and p65. In unstimulated cells, NF-*κ*B is in the cytoplasm bound to the inhibitor-kappa B (I*κ*B) as an inactive cytoplasmic precursor [7]. Extracellular stimuli could activate I*κ*B kinase (IKK), which leads to I*κ*B phosphorylation and degradation [8]. Subsequently, NF-*κ*B is liberated and translocates into the nucleus, where it actively regulates the transcription of a wide variety of reporter genes [9]. NF-*κ*B is thought to be a critical mediator of physiological and pathological processes including cell survival, proliferation, apoptosis, tumorigenesis, and inflammation [10]. NF-*κ*B has been reported to link inflammation with tumor progression and plays a functional role in inflammation and tumorigenesis [11]. NF-*κ*B is activated by a variety of stimuli including growth factors, cytokines, inflammatory agents, pharmacological agents, carcinogens, and stress.

Immunohistochemical analysis of urinary bladder tumor samples from 140 patients showed a strong correlation between cyclooxygenase 2 (COX-2) and nuclear NF-*κ*B immunoreactivity [12]. COX-2 is NF-*κ*B target gene [10]. In our previous study, COX-2 expression in bladder urothelium increased in DMA^V^ treated rats. Despite the critical importance of NF-*κ*B in cancer, the function of NF-*κ*B in urothelium of DMA^V^ treated rats remains poorly defined. In the present study, NF-*κ*B signal pathways in bladder epithelial cells of subchronic exposure to DMA^V^ rats were investigated to focus on the effects of DMA^V^ on NF-*κ*B signal pathways. In addition, inflammatory factors expressions in urothelium and secretion in urine of rats were also analyzed.

## 2. Materials and Methods

### 2.1. Chemicals

DMA^V^ (purity 99%) was purchased from Sangon Biotech (Shanghai, China). Trizol solution was from Invitrogen (Carlsbad, CA, USA). Real-Time PCR test kits were purchased from TaKara Biotechnology (Dalian, China). Polyclonal antibodies against IKK*α*, IKK*β*, phospho-p65, phospho-p50, and TGF-*β* were obtained from Sangon Biotech Co., Ltd. (Shanghai, China). IL-1*β* ELISA kits were obtained from Dakewe Bio-Engineering Limited Company (Shenzhen, China). All chemicals used in the study were of analytical grade.

### 2.2. Animals and Treatment

60 female healthy weanling SPF-grade Wistar rats (40–50 g) were obtained from Experimental Animal Center of China Medical University (China). After rats were acclimatized to the environmental conditions for one week, they were randomly selected into three groups consisting 20 rats per group. Group I includes control rats receiving distilled water as drinking water. Group II includes experimental rats receiving drinking water containing 100 ppm DMA^V^. Group III includes experimental rats receiving drinking water containing 200 ppm DMA^V^. The rats had free access to the standard rodent diet supplied by Experimental Animal Center of China Medical University and drinking water ad libitum. During the course of treatment, rats were observed daily for clinical signs, and daily water consumption and body weight gain were recorded periodically. All of the rats were kept in ventilated cages at 23–27°C, with 55–60% humidity and 12/12 h light/dark cycles. At last exposure week, 10 rats per group were placed in metabolic cages and 24 h urine samples were collected and frozen at −80°C until analyzed. After 10 weeks, exposure was stopped and rats were sacrificed under 10% chloral hydrate anesthesia. Within two minutes of the death of the rat, the bladders of 30 rats (10 rats each group) were ligated, rinsed, and then filled with cold Trizol solution for ten minutes. The cell lysate, containing urinary bladder urothelial cells, was aspirated for RNA extraction. The other bladders were fixed in 10% buffered paraformaldehyde and were cut longitudinally into strips, routinely embedded in paraffin for immunohistochemical analysis. The experimental procedure used in this study met the guidelines of the Animal Care and Use Committee of the China Medical University.

### 2.3. Quantitative Reverse Transcription-PCR (qRT-PCR) Analysis

Total RNA was extracted from the Trizol reagent of bladder epithelia according to the supplier's recommendations. The purity of each RNA sample was assessed by measuring absorbance at 260 and 280 nm and calculating the *A*_260_/*A*_280_ ratio. cDNA was synthesized using a reverse transcription reaction by the Easy RT-PCR kit according to the manufacturer's protocol. qRT-PCR reactions were performed using ABI 7500 real-time detection system (Applied Biosystems, Foster City, CA, USA) to determine the levels of mRNA. The primers for* IKKα*,* IKKβ*,* p65*,* p50*, and* IκBα* genes were synthesized by Sangon Biotech (Shanghai, China), and primer sequences are listed in Table 1. Thermocycling was performed in a final volume of 25 *μ*L containing 5 *μ*L of cDNA sample, 2 *μ*L of the primers, and 18 *μ*L of SYBR green PCR master mix (TaKara Biotechnology, Dalian, China). Fold changes for each gene expression were calculated using the Delta Ct method normalizing to *β-actin* expression for each sample.

### 2.4. Immunohistochemical Analysis

The bladder tissues in 10% paraformaldehyde were embedded in paraffin and then cut into 5 *μ*m sections on glass slides. Sections were deparaffinized, rehydrated, and subjected to a sequence of incubation steps starting with blocking endogenous peroxidase with 3% hydrogen peroxide at room temperature for 10 min. After microwave irradiation in 0.1 M PBS buffer for 10 minutes for epitope recovery, blocking buffer (5% bovine serum albumin, BSA) was added to each section and incubated at room temperature for 20 min. Then sections were incubated with polyclonal antibodies against IKK*α*, IKK*β*, p-p65, p-p50, and TGF-*β* in a humidity chamber at 37°C for 30 min. Subsequently, the slides were rinsed with PBS and incubated with secondary antibodies containing horseradish peroxidase at 37°C for 30 min. The slides were then washed in PBS and incubated with SABC reagent at 37°C for 30 min. After this treatment, the slides were stained with 3.3′-diaminobenzidine tetrahydrochloride (DAB) and then counterstained with hematoxylin. The immunohistochemical stainings were viewed and captured with a light microscope (Olympus bx51, Japan) at 200 or 400x magnification. The immunointensities of IKK*α*, IKK*β*, phospho-p65, phospho-p50, and TGF-*β* were measured with an image analyzer (Meta Morph, UIC, USA) by measuring the integrated optical density average (IOD) at five randomly selected fields. The positive rates of phospho-p65 and phospho-p50 expressions in nuclei were calculated by dividing the number of positive nuclei by the total number of nuclei counted, and the results were expressed as percentages (%).

### 2.5. ELISA Test

IL-1*β* in urine was measured using an ELISA kit according to the instructions of the manufacturer. The concentration of IL-1*β* was calculated according to the standard curve of the ELISA kits and expressed as pg/mL.

### 2.6. Statistical Analysis

Data were analyzed with SPSS for Windows, version 13.0. Differences between groups were statistically analyzed by LSD or Dunnett's T3 after one-way analysis of variance (ANOVA). The results were expressed as mean ± SD of number of experiments. *p* value < 0.05 was designated as statistically significant.

## 3. Results

### 3.1. General Observation

**Figure 1:**
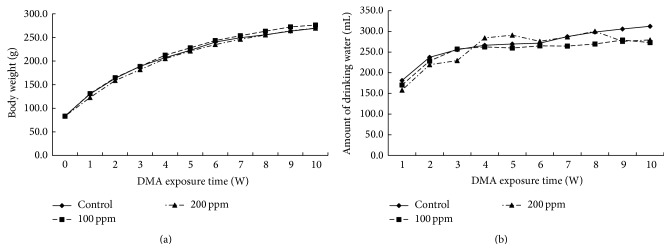
Body weight and real amount of drinking water of rats. The linear charts represent (a) body weight (g) and (b) amount of drinking water (mL) of rats after being exposed to 100 or 200 ppm DMA^V^ in drinking water from the first week to tenth week.

All rats were observed once daily with detailed evaluation during the study period. Administration of DMA^V^ (100 and 200 ppm) did not disturb food intake and body weight gain (Figure 1(a)). There was a little decrease of water consumption in 100 and 200 ppm DMA^V^ treated rats at 9th and 10th weeks as compared with control rats, but there were no significant differences in water consumption between the groups (Figure 1(b)).

### 3.2. mRNA Expressions of NF-*κ*B Signaling Molecules in the Bladder Epithelium of Rats Exposed to DMA^V^

**Figure 2:**
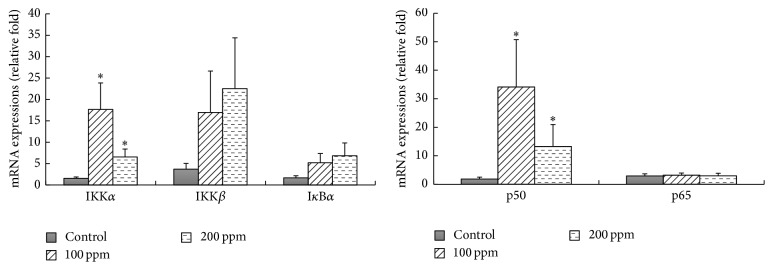
Effects of DMA^V^ on the mRNA expressions of* IKKα*,* IKKβ*,* p65*,* p50*, and* IκBα* of bladder epithelium in rats.* IKKα*,* IKKβ*,* p65*,* p50*, and* IκBα* mRNA levels of bladder epithelium in rats were measured by qRT-PCR. Bars were presented as mean ± SD. ^*∗*^ *p* < 0.05 compared to the control group.

The effect of DMA^V^ on the mRNA expressions of NF-*κ*B signaling molecules in the bladder epithelium of rats was depicted in Figure 2. The treatment with 100 and 200 ppm DMA^V^ for 10 weeks dramatically increased the mRNA levels of NF-*κ*B* p50* and* IKKα* in the bladder epithelium of rats compared to the control group (*p* < 0.05). Although there was a dose-dependent increase tendency for* IKKβ* mRNA expression, there were no statistical differences between DMA^V^ treated rats and control rats. Significant changes for NF-*κ*B* p65* and* IkBα* mRNA expressions were not seen.

### 3.3. Immunoreactivities for NF-*κ*B Signaling Molecules in the Bladder Epithelium of Rats Exposed to DMA^V^

**Figure 3:**
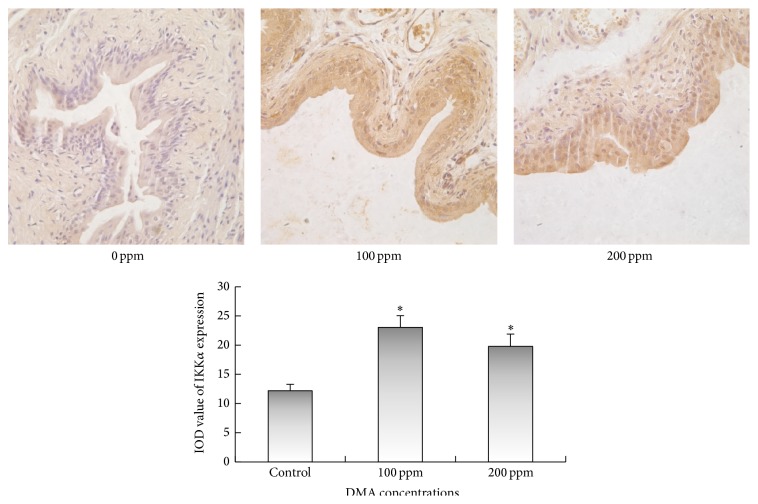
IKK*α* immunohistochemical staining of bladder epithelium in DMA^V^ treated rats. IKK*α* expressions were presented in brown in cytoplasm. The panel was a graphical representation of the immunointensities of IKK*α*. Bars were presented as mean ± SD. ^*∗*^ *p* < 0.05 compared to the control group.

**Figure 4:**
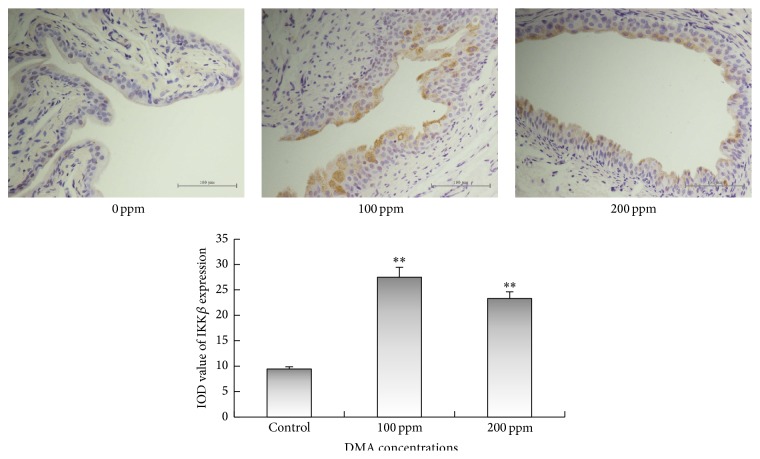
IKK*β* immunohistochemical staining of bladder epithelium in DMA^V^ treated rats. IKK*β* expressions were presented in brown in cytoplasm. The panel was a graphical representation of the immunointensities of IKK*β*. Bars were presented as mean ± SD. ^*∗∗*^ *p* < 0.01 compared to the control group.

**Figure 5:**
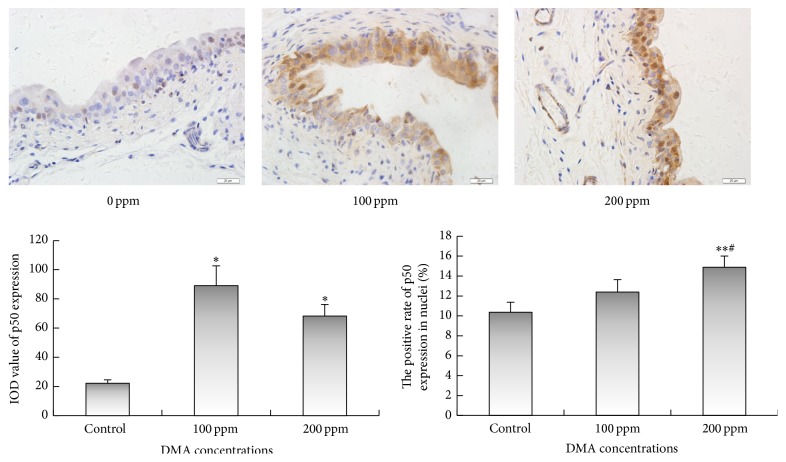
p50 immunohistochemical staining of bladder epithelium in DMA^V^ treated rats. p50 expressions were presented in brown in cytoplasm and nucleus. The panel was a graphical representation of the immunohistochemical staining of p50. Bars were presented as mean ± SD. ^*∗*^ *p* < 0.05 and ^*∗∗*^ *p* < 0.01 compared to the control group. ^#^ *p* < 0.05 compared to 100 ppm DMA^V^ treated group.

**Figure 6:**
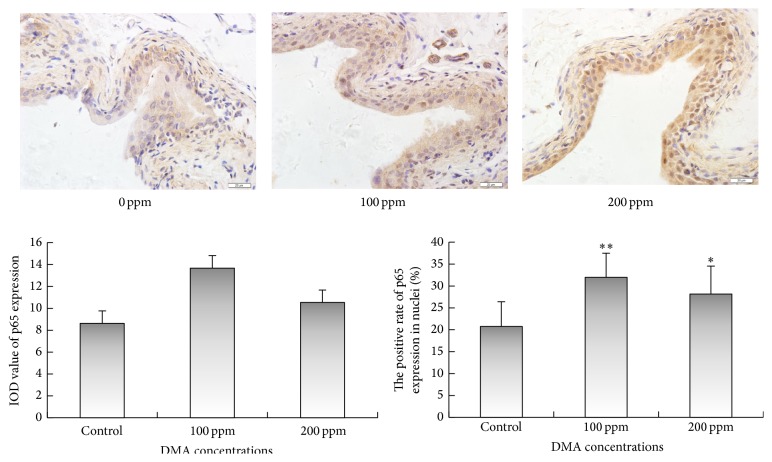
p65 immunohistochemical staining of bladder epithelium in DMA^V^ treated rats. p65 expressions were presented in brown in cytoplasm and nucleus. The panel was a graphical representation of the immunohistochemical staining of p65. Bars were presented as mean ± SD. ^*∗*^ *p* < 0.05 and ^*∗∗*^ *p* < 0.01 compared to the control group.

Immunohistochemical examinations revealed that 100 or 200 ppm DMA^V^ administration caused significant increase in the immunoreactivities of IKK*α* and IKK*β* in the cytoplasm of rat urothelial cells (Figures 3 and 4). Immunoreactivities of phospho-NF-*κ*B p50 in nucleus and cytoplasm displayed significant increase in DMA^V^ treated rat bladder epithelium (Figure 5). Nuclear phospho-NF-*κ*B p65 immunoexpression increased in 100 and 200 ppm DMA^V^ treated rat bladder epithelium and levels of phospho-NF-*κ*B p65 immunoexpression in cytoplasm did not increase in DMA^V^ treated rats compared with control rats (Figure 6).

### 3.4. DMA^V^ Increased TGF-*β* Expression in the Bladder Epithelium and Promoted Urinary IL-1*β* Secretion in Rats

**Figure 7:**
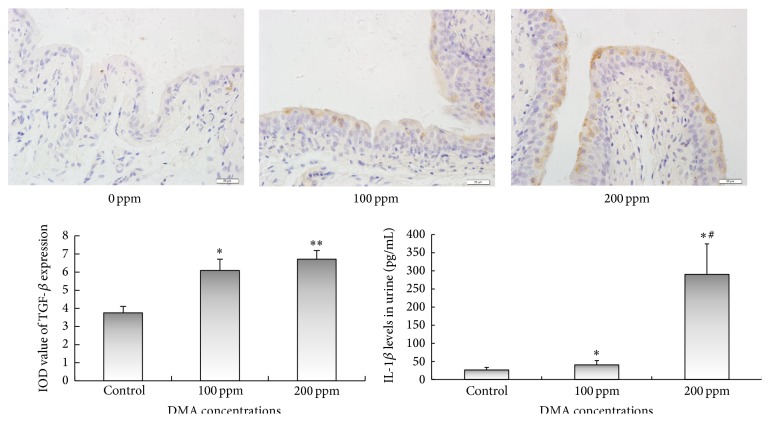
TGF-*β* immunohistochemical staining of bladder epithelium and urinary IL-1*β* levels in DMA^V^ treated rats. TGF-*β* expressions were presented in brown in cytoplasm. The panel was a graphical representation of the immunointensities of TGF-*β*. The concentrations of IL-1*β* in urine were measured by ELISA kits and bars were presented as mean ± SD. ^*∗*^ *p* < 0.05 and ^*∗∗*^ *p* < 0.01 compared to the control group.

In our previous study, 200 ppm DMA^V^ treated rats exhibited significantly increased inflammatory factor TGF-*β*1 level in urine compared to the control rats [5]. In the present immunohistochemical localization analysis, TGF-*β* expression was elevated in 100 and 200 ppm DMA^V^ treated rat bladder epithelium. In addition, 200 ppm DMA^V^ treatment also increased IL-1*β* secretion in urine of rats. These data suggest that DMA^V^ stimulates the production of proinflammatory cytokines in rat bladder; see Figure 7.

## 4. Discussion

Inorganic arsenic is a known human carcinogen and undergoes metabolic methylation in mammals and is metabolized to DMA^V^. However, DMA^V^ has been demonstrated to be a bladder carcinogen in rats [3, 13] and also enhanced bladder carcinogenesis when administered in the drinking water after treatment with N-butyl-N-(4-hydroxybutyl) nitrosamine, a known bladder carcinogen [14]. Inorganic arsenic likely has multiple mechanisms of carcinogenic action [4] and dysregulates some signal pathways through transcriptional and nongenomic mechanisms [15–17]. Few studies were reported on molecular mechanisms of DMA^V^ carcinogenesis. The transcriptional factor NF-*κ*B is activated in a range of human cancers and promotes tumorigenesis via regulation of target gene expression [18, 19]. NF-*κ*B is widely considered to play a major role in tumor development by promoting cell survival, proliferation, angiogenesis, and metastasis [10, 19]. It has been demonstrated that inorganic arsenic could activate NF-*κ*B signaling pathway [20, 21]. The effects of DMA^V^ on NF-*κ*B signaling pathway in bladder cells remain unclear. In the present study, we observed that expressions of members of the NF-*κ*B signaling pathway and IKK*α*, IKK*β*, p50, and p65 were increased in bladder epithelium of rats after exposure to DMA^V^.

NF-*κ*B activation was attributed to the phosphorylation of I*κ*B protein by IKK kinase. The activation of IKK kinase results in I*κ*B polyubiquitination and subsequent degradation by the 26S proteasome [22]. After that, the NF-*κ*B p50 and p65 subunits in cytoplasm activate and subsequently translocate into nucleus. Our study showed that 100 and 200 ppm DMA^V^ treatment in drinking water significantly increased* IKKα* and* IKKβ* mRNA levels and protein expressions in bladder epithelium of rats, which indicated DMA^V^ activated IKK kinase. In DMA^V^ exposure rat bladder epithelium, NF-*κ*B immunolocalization displayed the fact that p65 protein expression increased in the nucleus, whereas cytoplasmic p65 immunoexpression did not increase. p50 protein expression increased in both the nucleus and cytoplasm in DMA^V^ exposure rat bladder epithelium. Generally, nuclear immunoreactivity is regarded as a surrogate marker for the activated NF-*κ*B protein. The urothelial lining of the bladder is a responsive epithelial tissue with the ability to react to a variety of stimuli [23]. Thus, we thought that DMA^V^ led to the activation of the NF-*κ*B pathway in rat bladder epithelium.

NF-*κ*B was initially characterized as a central regulator in inflammatory and immune responses. It could connect chronic inflammation and tumorigenesis and was a potential molecular bridge between inflammation and cancer [24, 25]. NF-*κ*B is induced by various cell stresses including growth factors, cytokines, and oxidative stress [26] and in turn regulates numbers of genes encoding proteins involved in immune and inflammatory responses, such as cytokines, growth factors, immune receptors [27]. Inflammation has been thought to contribute to the development of cancer. Within the tumor microenvironment, certain inflammatory mediators often play a fundamental role in regulating tumor subpopulation expression [28]. Proinflammatory cytokines, IL-1*β* and IL-8, regulated by NF-*κ*B caused inflammatory responses and were critical components in the tumorigenesis pathway [28]. IL-1*β* was activated and amplified by NF-*κ*B via a positive amplifying loop [29, 30]. It has been reported that activated NF-*κ*B in cancer stem cells (CSCs) can promote a proinflammatory environment, inhibit apoptosis, and stimulate cell proliferation [31, 32]. Active NF-*κ*B translocates to the nucleus and binds to target proinflammatory genes, inducing transcription of cytokines, including IL-1*β* [33]. On the other hand, cytokines released from the tumor microenvironment could elevate NF-*κ*B activity [34]. TGF-*β* activated kinase, TAK1, was shown to mediate responses to cytokines TNF-*α* or IL-1 and directly phosphorylate the IKK complex that promotes activation of NF-*κ*B [35–37]. TGF-*β* induces NF-*κ*B activation through phosphorylation and activation of TAK1 in head and neck squamous cell carcinoma. TAK1, as an upstream mediator of IKK*α/β* phosphorylation and activation, leads to phosphorylation and degradation of the NF-*κ*B inhibitor I*κ*B*α* and induces nuclear translocation and transactivation of NF-*κ*B [38]. In the present study, we observed that DMA^V^ increased TGF-*β* expression in bladder epithelium and IL-1*β* secretion in urine of rats. It is widely accepted that TGF-*β* and IL-1 are powerful signaling molecules and involve many cellular processes, and their altered expression profiles are associated with various pathologies [39]. Overexpression of TGF-*β* in the tumor microenvironment was often observed [40] and TGF-*β* was one of the key growth factors involved in driving epithelial-mesenchymal transition (EMT) [41]. IL-1*β* both induced inflammation by activating NF-*κ*B signaling [37] and also was controlled by NF-*κ*B signaling [42].

Our precious study found DMA elevated expressions of proliferation factors, such as PCNA, cyclin D1, and COX-2, in bladder epithelium [5]. The possible mechanism of DMA^V^ on bladder epithelium in this study was speculated because DMA^V^ activated NF-*κ*B signal pathway, which increased the secretion of TGF-*β* and IL-1*β* in rats. Furthermore, increased expressions of TGF-*β* and IL-1*β* might mediate activation of NF-*κ*B signal pathway to promote cell proliferation.

## Figures and Tables

**Table 1:** The sense and antisense primers used for qRT-PCR.

Genes	Gene number	Forward and reverse	Length
*IKKα*	NM_001107588.1	Forward: GAAGGCACAGTAACCCCTCC	297 bp
		Reverse: TCCGCAGGAAAGATGACCAC	

*IKKβ*	NM_053355.2	Forward: CTGTGCACGTCATTTGTGGG	146 bp
		Reverse: CAGTTAGGGAGAAAGGGCCG	

IkB*α*	NM_001105720.2	Forward: CTCAAGAAGGAGCGGTTGGT	184 bp
		Reverse: CCAAGTGCAGGAACGAGTCT	

*p65*	NM_199267.2	Forward: AACACTGCCGAGCTCAAGAT	163 bp
		Reverse: CATCGGCTTGAGAAAAGGAG	

*p50*	NM_001276711.1	Forward: AGAGCAACCGAAACAGAGAGG	141 bp
		Reverse: TTTGCAGGCCCCACATAGTT	

*β-actin*	NM_031144.3	Forward: TCACCCACACTGTGCCCATCTATGA	295 bp
		Reverse: CATCGGAACCGCTCATTGCCGATAG	

## References

[1] Yamamoto S., Konishi Y., Matsuda T. (1995). Cancer induction by an organic arsenic compound, dimethylarsinic acid (cacodylic acid), in F344/DuCrj rats after pretreatment with five carcinogens. *Cancer Research*.

[2] Wei M., Wanibuchi H., Morimura K. (2002). Carcinogenicity of dimethylarsinic acid in male F344 rats and genetic alterations in induced urinary bladder tumors. *Carcinogenesis*.

[3] Arnold L. L., Eldan M., Nyska A., van Gemert M., Cohen S. M. (2006). Dimethylarsinic acid: results of chronic toxicity/oncogenicity studies in F344 rats and in B6C3F1 mice. *Toxicology*.

[4] International Agency for Research on Cancer (IARC) (2004). Arsenic in drinking water. *Some Drinking Water Disinfectants and Contaminants, Including Arsenic*.

[5] Lin Z., Shengnan L., Fei W. (2015). Dimethylarsinic acid (DMA^V^) changed the expressions of proliferative related factors and secretion of inflammatory cytokines in rat bladder. *Journal of Applied Toxicology*.

[6] Levidou G., Saetta A. A., Korkolopoulou P. (2008). Clinical significance of nuclear factor (NF)-*κ*B levels in urothelial carcinoma of the urinary bladder. *Virchows Archiv*.

[7] Baldwin A. S. (1996). The NF-kappa B and I kappa B proteins: new discoveries and insights. *Annual Review of Immunology*.

[8] May M. J., Ghosh S. (1999). I*κ*B kinases: kinsmen with different crafts. *Science*.

[9] Dejardin E., Deregowski V., Chapelier M. (1999). Regulation of NF-*κ*B activity by I*κ*B-related proteins in adenocarcinoma cells. *Oncogene*.

[10] Karin M., Cao Y., Greten F. R., Li Z.-W. (2002). NF-kappaB in cancer: from innocent bystander to major culprit. *Nature Reviews Cancer*.

[11] Greten F. R., Eckmann L., Greten T. F. (2004). IKKbeta links inflammation and tumorigenesis in a mouse model of colitis-associated cancer. *Cell*.

[12] Kontos S., Sotiropoulou-Bonikou G., Kominea A., Melachrinou M., Balampani E., Bonikos D. (2010). Coordinated increased expression of cyclooxygenase2 and nuclear factor *κ*B is a steady feature of urinary bladder carcinogenesis. *Advances in Urology*.

[13] Wei M., Wanibuchi H., Yamamoto S., Li W., Fukushima S. (1999). Urinary bladder carcinogenicity of dimethylarsinic acid in male F344 rats. *Carcinogenesis*.

[14] Wanibuchi H., Yamamoto S., Chen H. (1996). Promoting effects of dimethylarsinic acid on *N*-butyl-*N*-(4-hydroxybutyl)nitrosamine-induced urinary bladder carcinogenesis in rats. *Carcinogenesis*.

[15] Liu S., Wang F., Yan L. (2013). Oxidative stress and MAPK involved into ATF2 expression in immortalized human urothelial cells treated by arsenic. *Archives of Toxicology*.

[16] Wang F., Liu S., Xi S. (2013). Arsenic induces the expressions of angiogenesis-related factors through PI3K and MAPK pathways in SV-HUC-1 human uroepithelial cells. *Toxicology Letters*.

[17] Ahlborn G. J., Nelson G. M., Ward W. O. (2008). Dose response evaluation of gene expression profiles in the skin of K6/ODC mice exposed to sodium arsenite. *Toxicology and Applied Pharmacology*.

[18] Gilmore T. D. (2006). Introduction to NF-*κ*B: players, pathways, perspectives. *Oncogene*.

[19] Chaturvedi M. M., Sung B., Yadav V. R., Kannappan R., Aggarwal B. B. (2011). NF-*κ*B addiction and its role in cancer: ‘one size does not fit all’. *Oncogene*.

[20] Huang S., Guo S., Guo F. (2013). CD44v6 expression in human skin keratinocytes as a possible mechanism for carcinogenesis associated with chronic arsenic exposure. *European Journal of Histochemistry*.

[21] Jiang R., Li Y., Xu Y. (2013). EMT and CSC-like properties mediated by the IKK*β*/I*κ*B*α*/RelA signal pathway via the transcriptional regulator, Snail, are involved in the arsenite-induced neoplastic transformation of human keratinocytes. *Archives of Toxicology*.

[22] Hseu Y.-C., Senthil Kumar K. J., Chen C.-S. (2014). Humic acid in drinking well water induces inflammation through reactive oxygen species generation and activation of nuclear factor-*κ*B/activator protein-1 signaling pathways: a possible role in atherosclerosis. *Toxicology and Applied Pharmacology*.

[23] Olsson L. E., Wheeler M. A., Sessa W. C., Weiss R. M. (1998). Bladder instillation and intraperitoneal injection of *Escherichia coli* lipopolysaccharide up-regulate cytokines and iNOS in rat urinary bladder. *Journal of Pharmacology and Experimental Therapeutics*.

[24] Holdenrieder S., Stieber P. (2004). Apoptotic markers in cancer. *Clinical Biochemistry*.

[25] Celec P. (2004). Nuclear factor *κ* B-molecular biomedicine: the next generation. *Biomedicine and Pharmacotherapy*.

[26] Karin M., Greten F. R. (2005). NF-kappaB: linking inflammation and immunity to cancer development and progression. *Nature Reviews Immunology*.

[27] Rehman M. U., Tahir M., Khan A. Q. (2013). Chrysin suppresses renal carcinogenesis via amelioration of hyperproliferation, oxidative stress and inflammation: plausible role of NF-*κ*B. *Toxicology Letters*.

[28] Bourguignon L. Y. W., Wong G., Earle C. A., Xia W. (2011). Interaction of low molecular weight hyaluronan with CD44 and toll-like receptors promotes the actin filament-associated protein 110-actin binding and MyD88-NF*κ*B signaling leading to proinflammatory cytokine/chemokine production and breast tumor invasion. *Cytoskeleton*.

[29] Ruiz-Deya G., Sikka S. C., Thomas R., Abdel-Mageed A. B. (2002). Potential role for the nuclear transcription factor NF-*κ*B in the pathogenesis of ureteropelvic junction obstruction. *Journal of Endourology*.

[30] Zhu T., Zhang L., Ling S. (2014). Scropolioside B inhibits IL-1*β* and cytokines expression through NF-*κ*B and inflammasome NLRP3 pathways. *Mediators of Inflammation*.

[31] Chefetz I., Holmberg J. C., Alvero A. B., Visintin I., Mor G. (2011). Inhibition of Aurora-a kinase induces cell cycle arrest in epithelial ovarian cancer stem cells by affecting NF*κ*B pathway. *Cell Cycle*.

[32] Alvero A. B., Chen R., Fu H.-H. (2009). Molecular phenotyping of human ovarian cancer stem cells unravels the mechanisms for repair and chemoresistance. *Cell Cycle*.

[33] Wang X.-C., Saban R., Kaysen J. H. (2000). Nuclear factor kappa B mediates lipopolysaccharide-induced inflammation in the urinary bladder. *Journal of Urology*.

[34] Ben-Neriah Y., Karin M. (2011). Inflammation meets cancer, with NF-*κ*B as the matchmaker. *Nature Immunology*.

[35] Jackson-Bernitsas D. G., Ichikawa H., Takada Y. (2007). Evidence that TNF-TNFR1-TRADD-TRAF2-RIP-TAK1-IKK pathway mediates constitutive NF-*κ*B activation and proliferation in human head and neck squamous cell carcinoma. *Oncogene*.

[36] Sakurai H., Miyoshi H., Toriumi W., Sugita T. (1999). Functional interactions of transforming growth factor *β*-activated kinase 1 with I*κ*B kinases to stimulate NF-*κ*B activation. *Journal of Biological Chemistry*.

[37] Takaesu G., Kishida S., Hiyama A. (2000). TAB2, a novel adaptor protein, mediates activation of TAK1 MAPKKK by linking TAK1 to TRAF6 in the IL-1 signal transduction pathway. *Molecular Cell*.

[38] Freudlsperger C., Bian Y., Contag Wise S. (2013). TGF-*β* and NF-*κ*B signal pathway cross-talk is mediated through TAK1 and SMAD7 in a subset of head and neck cancers. *Oncogene*.

[39] Gordon G. M., Ledee D. R., Feuer W. J., Fini M. E. (2009). Cytokines and signaling pathways regulating matrix metalloproteinase-9 (MMP-9) expression in corneal epithelial cellsy. *Journal of Cellular Physiology*.

[40] Cui W., Fowlis D. J., Bryson S. (1996). TGF*β*1 inhibits the formation of benign skin tumors, but enhances progression to invasive spindle carcinomas in transgenic mice. *Cell*.

[41] Kim H., Choi J.-A., Kim J.-H. (2014). Ras promotes transforming growth factor-*β*(TGF-*β*)-induced epithelial-mesenchymal transition via a leukotriene B4 receptor-2-linked cascade in mammary epithelial cells. *The Journal of Biological Chemistry*.

[42] Catley M. C., Chivers J. E., Cambridge L. M. (2003). IL-1beta dependent activation of NF-kappaB mediates PGE2 release via the expression of cyclooxygenase-2 and microsomal prostaglandin E synthase. *FEBS Letters*.

